# IL2RG knockout mitigates polycystic ovary syndrome pathogenesis by transitioning pyroptosis to apoptosis through the GSDME pathway

**DOI:** 10.1186/s13048-025-01774-4

**Published:** 2025-08-19

**Authors:** Sailing Chen, Han Lu, Yingfen Ying, Haitao Xi, Jingyi Zheng, Chaochao Gong, Shaowei Wang, Yunbin Tang, Junzhao Zhao, Ren-shan Ge, Xiaoheng Li

**Affiliations:** 1 Department of Obstetrics and Gynecology, the Second Affiliated Hospital, Yuying Children’s Hospital of Wenzhou Medical University, Wenzhou, 325027 Zhejiang China; 2 Department of Anesthesiology and Perioperative Medicine, The Second Affiliated Hospital and Yuying Children’s Hospital of Wenzhou Medical University; Key Laboratory of Pediatric Anesthesiology, Ministry of Education, Wenzhou Medical University; Key Laboratory of Precision Anesthesiology of Zhejiang Province, Wenzhou Medical University, Wenzhou, 325027 Zhejiang China; 3 Department of Obstetrics and Gynecology, Reproductive Medicine Center, The Second Affiliated Hospital of Wenzhou Medical University, 325027 Wenzhou, China

**Keywords:** Polycystic ovary syndrome, IL2RG, Gasdermin E, Apoptosis, Granulosa cells

## Abstract

**Background:**

Polycystic ovary syndrome (PCOS) is a common endocrine disorder with significant impacts on women’s reproductive health. Interleukin (IL)-2 receptor subunit gamma (IL2RG), a common receptor for IL-2, IL-4, IL-7, IL-9, IL-15 and IL-21, has been shown to interrupt estrous cycle, yet its role in PCOS remains unclear.

**Results:**

The evaluated expression levels of IL2RG in human granulosa cells (GCs) and IL2RG-dependent cytokines (IL-2, IL-4, IL-15) in follicular fluid were demonstrated in 18 PCOS patients and 22 control subjects. The positive correlation between IL2RG and PCOS was analysed through GEO databases. In vivo, we found that in the PCOS model (6 mg/100 g body-weight of dehydroepiandrosterone subcutaneous injection for 21 days), the ovarian index, testosterone, glucose, and LH/FSH levels were elevated, and the estrous cycle was disrupted. Knocking down IL2RG (KO) restored the above levels. However, in the dehydroepiandrosterone-treated group, these levels did not recover even after IL2RG was knocked-down. The decreased expression of Gasdermin E (GSDME) but increased caspase-3 level in IL2RG KO rats compared with WT were found. In vitro, knockdown of IL2RG by siRNA inhibited caspase-3-mediated GSDME cleavage in testosterone-induced KGN cells. Furthermore, the caspase-3 inhibitor Z-DEVD-FMK and the caspase-1 inhibitor Belnacasan alleviated pyroptosis in testosterone-induced KGN cells by lactate dehydrogenase test, fluorescence assay, and flow cytometry.

**Conclusions:**

IL2RG is expressed higher in PCOS patients, exerting an inhibitory effect on caspase-3-mediated GSDME cleavage upon knockdown. However, the knockout of IL2RG led to the conversion of GSDME-mediated pyroptosis into apoptosis. This study explores the function of IL2RG and provides insights for therapeutic targets in PCOS.

**Supplementary Information:**

The online version contains supplementary material available at 10.1186/s13048-025-01774-4.

## Introduction

Polycystic ovary syndrome (PCOS) is a common endocrine disorder affecting women of reproductive age, with a prevalence ranging from 10 to 13%. Characterized by chronic anovulation and hyperandrogenism, PCOS is associated with physiological inflammation in the female reproductive tract during ovulation, menstruation, and labor [1, 2]. Persistent low-level inflammation in PCOS patients contributes to excessive androgen production by polycystic ovaries and increases the risk of cardiovascular complications [3].

Interleukin (IL)-2 family members bind to their respective receptors and share a critical subunit receptor, the IL-2 receptor subunit gamma (IL2RG). IL2RG is a shared subunit for several ILs, including IL-2, IL-4, IL-7, IL-9, IL-15, and IL-21, which are crucial for the development and survival of immune cell subsets like T and NK cells [4, 5]. Although IL-2 family members play a critical role in development and maintenance of the immune system, they also have roles in other systems such as reproduction. IL-15, one of the ligands of IL2RG, had been reported to contribute to PCOS by affecting the inflammatory status and steroid hormone production of granulosa cells (GCs) [6]. Additionally, studies on IL2RG knockout (KO) mice have shown irregular estrous cycles and placenta or fetal growth [7, 8]. These observations suggested that IL2RG participates in ovarian function. But, whether IL2RG contributes to PCOS and what are the specific regulatory mechanisms remain unknown.

Dysregulation of pyroptosis, a form of programmed cell death characterized by inflammasome activation, has been implicated in the pathogenesis of sterile inflammatory diseases [9]. It is initiated by the cleavage of gasdermins, caspases, and granzymes. This cleavage leads to pore formation in cellular membranes, cell lysis, and the release of pro-inflammatory molecules [10, 11]. Specifically, the inflammasome complex plays a central role in this process. It recruits and activates caspase-1 (CASP1) or CASP3, which then convert pro-IL-1β and pro-IL-18 into their active forms [11–13].

Recent studies have found that hyperandrogen exposure induced ovarian dysfunction and fibrosis by activating NLRP3 (the nucleotide-binding oligomerization domain leucine-rich repeat and pyrin domain-containing protein 3). It plays a crucial role in the innate immune system, particularly in the formation of the NLRP3 inflammasome. The NLRP3 inflammasome is a multiprotein complex that, upon activation, leads to the processing and release of pro-inflammatory cytokines such as IL-1β and IL-18, which are important for the body’s response to various stimuli including NLRP3 inflammasome in PCOS mice [14]. As one of key gasdermins, GSDME is highly expressed in pyroptosis, and its presence often results in CASP3 activation by chemotherapy drugs, leading to pyroptotic characteristics due to the faster progression of pyroptosis compared to apoptosis [15]. The presence of GSDME can switch apoptosis to pyroptosis, depending on the context and the activation of CASP3 [16]. However, whether GSDME is involved in the pathogenesis of PCOS and acts as a switch that converts apoptosis into pyroptosis under PCOS conditions remains unclear.

In this study, we demonstrated that elevated concentrations of certain IL2RG-dependent cytokines in women with PCOS. We also investigated the correlation between IL2RG and GSDME and explored the impact of IL2RG on GC biological activities. Our findings indicate that suppression of IL2RG effectively alleviates inflammation in PCOS patients by reducing pyroptosis. Understanding the relationship between IL2RG and GSDME in PCOS could provide new insights into the pathogenesis of this condition.

## Methods

### Study subjects and sample collection

The diagnosis of PCOS was based on the Rotterdam criteria. Control group comprised women seeking treatment for tubal infertility or male factor issues, who had normal ovarian reserve. Excluded from the study were individuals with conditions such as endometriosis, ovarian cancer, or other endocrine disorders. Follicular fluid (FF) samples were collected from 18 PCOS patients and 22 controls for cytokine analysis. Clinical characteristics of the participants were detailed in Table 1. FF samples were immediately centrifuged at 550 ×g for 15 min, and the resulting supernatants were stored at -80 °C until further analysis. GCs were isolated through Percoll gradient centrifugation [17].

### Data analysis

The microarray datasets GSE34562, GSE98421, GSE216609, and GSE106724 were retrieved from the GEO database. To ensure data compatibility, raw data were processed using the GEO2R tool (https://www.ncbi.nlm.nih.gov/geo/geo2r/). Bioinformatics tools like the STRING database (https://string-db.org/) were used. Relevant genes (such as *GSDME* and *IL2RG*) were input and a protein-protein interaction network, which demonstrates the interaction relationships among gene products, was plotted. Data of *GSDME* (also called DFNA5) and *IL2RG* genes in various cancer types (https://compbio.cn/timer2) were retrieved. Spearman’s correlation analysis was conducted, significance by *P*-value was determined, and results with color-coding and symbols were visually represented. DFNA5 (GSDME) and IL2RG data from GEPIA (http://gepia.cancer-pku.cn/detail.php?clicktag = correlation) were extracted and Spearman’s correlation analysis to show their relationship was performed.

### PCOS modeling

Sprague-Dawley female rats (6–8 weeks old) were obtained from Vital River Laboratory Animal Technology Co Ltd. (Beijing, China). IL2RG knockout (KO) rats were sourced from Biocytogen (Beijing, China). The IL2RG KO rats were generated by Biocytogen using CRISPR/Cas9 technology to target exon 2 of the IL2RG gene. By removing a portion of exon 2 (Δ14/Δ14), the normal *Il2rg* sequence is disrupted, resulting in a non-functional protein and creating an immunodeficient rat model suitable for studying immune-related diseases and humanized research applications. The rats were housed in pairs per cage under a 12-h light/dark cycle, with controlled temperature and humidity. The rats were randomly assigned to four groups: wild type (WT) rats treated with corn oil (WT + C), WT rats treated with dehydroepiandrosterone (DHEA) (WT + D), KO rats treated with corn oil (KO + C), and KO rats treated with DHEA (KO + D) (*n* = 7–8 per group). To establish the PCOS model, we administered DHEA subcutaneously, as previously described in the literature [18, 19]. This model is characterized by elevated androgen levels, increased LH/FSH ratio, cystic follicle development, and disrupted estrous cycles, mimicking key features of human PCOS [20]. To induce the PCOS phenotype, rats in the DHEA group were subcutaneously injected with 6 mg/100 g bodyweight DHEA (CAS. 53-43-0, Aladdin, Shanghai, China) daily for 21 consecutive days. Rats in the control group received the same volume of corn oil injections.

### Histology

Five litters per group and one rat per litter were randomly selected for histological analysis. The ovaries were fixed in Bouin’s fluid and then sectioned into 5 μm thick slices for histological examination. All sections were stained with hematoxylin and eosin (H&E). Immunohistochemical (IHC) staining for IL2RG was performed according to the manufacturer’s instructions [21]. The sections were incubated overnight at 4 °C in a humid chamber with a rabbit antibody against IL2RG (ab273023, Abcam, USA) diluted at a ratio of 1:100. Observations were conducted using a Nikon Eclipse 80i microscope (Nikon, Tokyo, Japan). The slides were digitally scanned using the Nanozoomer-XR scanner (Hamamatsu, Japan) at a scanning speed of 0.23 μm/pixel to obtain digital images.

### Serum hormone ELISA measurement

Serum level of estradiol (E2) and total testosterone (TT) were measured by an Immunoassay Analyzer using Immulite2000 E2 kit (Sinopharm Group Medical Supply Chain Services Co, Hangzhou, China) and Immulite2000 testosterone kit, respectively. Serum luteinizing hormone (LH), follicle-stimulating hormone (FSH), anti-Müllerian hormone (AMH), and sex hormone-binding globulin (SHBG) levels were all determined using commercial human-specific ELISA kits (Solarbio, Beijing, China) following the manufacturer’s instructions. Free androgen index (FAI) = TT/SHBG*100%.

### Glucose tolerance tests

Rats were fasted for 12 h before undergoing glucose tolerance tests (GTT). Glucose levels were measured by tail vein blood sampling using the AccuChek Performa blood glucose analyzer (Roche Diagnostics, Indianapolis, IN, USA). The rats were intraperitoneally injected with D-glucose (2 g/kg body weight) for GTT after measuring glucose levels, and tail samples were collected at 0, 15, 30, 60, 90, and 120 min post-injection for glucose level detection.

### Estrous cycle monitoring

Typically, a regular reproductive cycle in female rats consists of four distinct phases (proestrus, estrus, metestrus, and diestrus), which can be identified by examining the cellular composition in vaginal smears. We collected vaginal samples from all mice participants and employed Gram Staining (Solarbio, China) to analyze their estrous cycles under a microscope (Nikon Eclipse 80i, Tokyo, Japan).

### Western blot analysis

Three ovaries were taken from each of three randomly selected litters in each group for western blot analysis. The protein concentration was determined using a bicinchoninic acid protein assay kit (Beyotime Biotechnology, China). The protein membranes were blocked with skim milk in TBS-T for 1 h and incubated overnight at 4 °C with the primary antibody in blocking buffer. The membrane was incubated by following antigens: anti-CASP3 [9662, Cell Signaling Technology (CST), Danvers, MA, USA, 1:1000), anti-IL2RG (ab273023, Abcam, 1:1000), β-Actin (ACTB, 4970, CST, 1:1000), and glyceraldehyde-3-phosphate dehydrogenase (GAPDH, AF7021, Affinity Biologicals, Ancaster, Ontario, Canada, 1:1000). The membrane was then incubated with HRP-conjugated anti-rabbit IgG secondary antibody (GAR007, Multi Sciences, Hangzhou, China, 1:2000) for 2 h and chemiluminescence images were developed using a Thermo Scientific Pierce ECL chemiluminescence kit (Pierce, USA). The gray scale intensity of the proteins was calculated using Bio-Rad Image Lab software (Hercules, CA, USA).

### Cell culture and treatment

KGN (human ovarian granulosa cell tumor) cell line which is a good model for studying GC biology [22] was obtained from Fuheng Biology (Shanghai, China) and cells were cultured in DMEM/F12 medium (Gibco, Grand Island, NY, USA) supplemented with 10% fetal bovine serum and 1% penicillin-streptomycin in a humidified incubator at 37°C with 5% CO_2_. To evaluate the effects of hyperandrogenism on KGN cells, cells were treated with 10 µM testosterone (T) for 24 hours. siRNA against *IL2RG* (siIL2RG) and its negative control were transfected using Lipofectamine 3000 (Life Technologies, Gaithersburg, MD, USA). The target sequences of the siRNAs were as follows: siIL2RG-1: forward: 5’-GGAACAACAGAUUCUUGAATT-3’ and reverse: 5’-UUCAAGAAUCUGUUGUUCCTT-3’; siIL2RG-2: forward: 5’-GGUACAAGAACUCGGAUAATT-3’ and reverse: 5’-UUAUCCGAGUUCUUGUACCTT-3’; siIL2RG-3: forward: 5’-CCUGGAGUGGUGUGUCUAATT-3’ and reverse: 5’-UUAGACACACCACUCCAGGTT-3’. CASP3 inhibitor, Z-DEVD-FMK (ZDF, GlpBio, Montclair, CA, USA), was treated at a concentration of 20 µM. Caspase-1 inhibitor, Belnacasan (VX-765, MCE, New Jersey, USA), was treated at a concentration of 10 µM.

### Scanning electron microscopy (SEM)

The KGN cells were first fixed in 2.5% glutaraldehyde and then dehydrated through a gradient series of ethanol solutions (30%, 50%, 70%, 100%, and 100%) for 10 min each. After dehydration, the samples were transferred to a supercritical dryer for critical point drying. Once the drying process was complete, the samples were removed from the dryer, affixed to a sample table with conductive tape, coated with gold, and examined using a scanning electron microscope (SU8020, Hitachi, Japan). For conventional sample preparation, the samples were directly affixed to the sample table with conductive tape, coated with gold, and examined.

### Quantitative Real-Time polymerase chain reaction (qRT-PCR)

The levels of specific mRNAs (*IL2RG*) in KGN cells and the internal control mRNA *ACTB* and positive control *GAPDH* were examined using qRT-PCR. Ovarian tissue was subjected to RNA extraction using a Trizol kit (Vazyme, Nanjing, China), following a previously established protocol [21]. Reverse transcription was performed using a reverse transcription kit and random primers (Vazyme). qRT-PCR was conducted using the SYBR Green qPCR kit (Bio Smile Co., China). The Ct values from each qRT-PCR run were recorded and used to generate a standard equation for the standards. The relative mRNA levels were normalized to *ACTB* for further analysis. Specific sequence primers were designed as follows: *IL2RG*: forward: 5’-GTGCAGCCACTATCTATTCTCTG-3’ and reverse: 5’-GTGAAGTGTTAGGTTCTCTGGAG-3’; *ACTB*: forward: 5’-CTCCATCCTGGCCTCGCTGT-3’ and reverse: 5’-GCTGTCACCTTCACCGTTCC-3’; *GAPDH*: forward: 5’-CTGCACCACCAACTGCTT-3’ and reverse: 5’-TTCTGGGTGGCAGTGATG-3’.

### Immunofluorescence

The death pattern was accurately characterized by adding propidium iodide (PI) and Fluorescein isothiocyanate (FITC)-Annexin V to the culture medium, PI is commonly used in flow cytometry and fluorescence microscopy to identify dead cells. Since it cannot pass through an intact cell membrane, it only stains cells with compromised membranes (such as dead or dying cells), allowing for the discrimination between live and dead cells in a sample. PI and FITC-Annexin V was gently mixed to avoid damaging lytic cell death. Cells were labeled with Annexin V and FITC-Annexin V (AP101, Lianke, China) and assessed at 0 to 24 h. Images were captured using the Operetta CLS™ high-content analysis system (UltraVIEW VOX, PerkinElmer, USA). At least three independent fields were imaged.

### Lactate dehydrogenase (LDH) assay

KGN cells were cultured in 96-well plates at 1 × 10⁴ cells/well for 24 h. The cell culture supernatants were detected using an LDH Cytotoxicity Assay Kit (C0016, Beyotime, China), following the manufacturer’s instructions. All groups were replicated three times.

### Flow cytometry

The cells were seeded at a density of approximately 70% before drug treatments. After harvesting, the cells were washed with cold phosphate buffered saline (PBS) and stained with FITC-labeled Annexin V and PI using the FITC Annexin V apoptosis kit (AP101, Lianke, China). The data were obtained using the CytoFLEX (Beckman Coulter) and analyzed with CytExpert software.

### Statistics

Assays were repeated three to four times. Data are presented as means ± SEM (standard error of the mean). Student’s t-test of two independent samples was used for data with normal distribution. Shapiro-Wilk test was used to test normality of small-size samples. When the data showed normal distribution, Pearson’s correlation analysis was used for correlation analysis. When the data did not show normal distribution, non-parametric test was used. The one-way ANOVA with Tukey’s multiple comparison post-hoc test was used for multiple groups. The post hoc statistical power value is expressed as “$”. Significant differences are indicated as *P < 0.05, **P < 0.01, and ***P < 0.001.

## Results

### Higher level of IL2RG in the granulosa cells of patients with PCOS

To assess the alteration of cytokine and IL2RG expression levels in women with PCOS, we recruited a total of 18 PCOS patients and 22 non-PCOS individuals, whose clinical characteristics are detailed in Table 1. In PCOS patients, the body mass index (BMI) and serum levels of LH, TT, AMH, infertility duration, and LH/FSH ratio were significantly elevated, while FSH and E2 levels were lower compared to the control group. Non-PCOS patients were matched with PCOS patients in terms of age and number of oocytes. A limitation of our study is the relatively small sample size in our human cohort (22 PCOS patients and 18 controls). But our study provides valuable preliminary evidence supporting the role of IL2RG in PCOS pathogenesis and highlights the potential of targeting this pathway for therapeutic intervention.

**Fig. 1:**
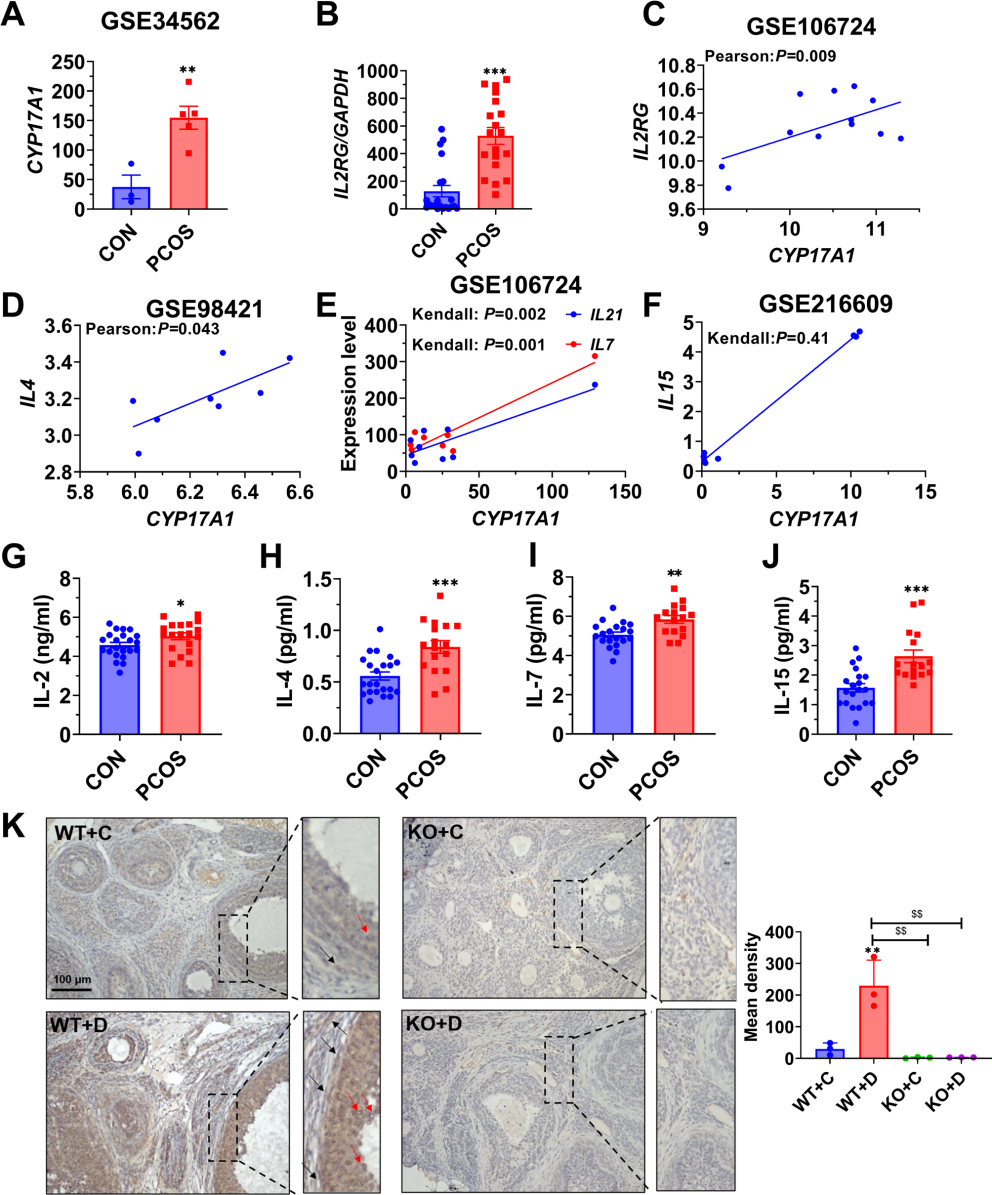
Expression of IL2RG and its dependent cytokines in the follicular fluid of PCOS patients. (**A**) Expression of *CYP17A1* in PCOS patients (GSE34562). (**B)** Expression of *IL2RG* in granulosa cells from PCOS patients was assessed using qRT-PCR, followed by statistical analysis using a t-test. (**C**) Correlation of relative expression of *IL2RG* with *CYP17A1* in cumulus granulosa cells of PCOS patients (GSE106724) as determined by Pearson analysis. (**D-F**) Correlation of relative expression of *CYP17A1* with *IL4* (GSE98421), *IL7* (GSE106724), *IL15* (GSE216609) and *IL21* (GSE106724). (**G-J**) IL-2, IL-4, IL-7 and IL-15 levels in follicular fluid of PCOS patients and controls measured by ELISA (PCOS *n* = 18, CON *n* = 22). (**K**) Mean pixel density of IL2RG expression in the ovary. IL2RG was stained in brown immunohistochemically. WT stands for wild type. Red arrow: granulosa cells expressing IL2RG; Black arrow: theca cells expressing IL2RG. The scale bars represent 100 μm in length. Data are given as mean ± SEM. *P < 0.05, **P < 0.01

CYP17A1/P450C17 (cytochrome P450 family 17 subfamily A member 17) is considered as the key enzyme responsible for major androgen production in PCOS [23]. Relative expression of CYP17A1 was positively correlation in cumulus granulosa cells of PCOS patients according to GSE34562 database which supported PCOS animal model (*P* = 0.0077, Fig. 1A). Additionally, higher expression levels of IL2RG were observed in PCOS by qRT-PCR (Fig. 1B). IL2RG is a common receptor for multiple cytokines such as IL-2, IL-4, IL-7, IL-9 and IL-15 [24]. Correlation analysis revealed a positive association between the concentration of IL-2, IL-4, IL-7, and IL-21 except IL-15 in FF and CYP17A1 expression in granulosa cells of patients with PCOS, as determined through bioinformatics analysis (Fig. 1C-F). According to the findings of ELISA assay, we found that IL-2, IL-4, IL-7 and IL-21 were significantly increased in PCOS group (Fig. 1G-J). The immunohistochemical staining of IL2RG in ovarian tissue revealed its expression in both granulosa and theca cells, as demonstrated by Fig. 1K which presents the mean pixel density measurements. In the WT + D group, IL2RG was highly expressed, whereas it was very low in the KO group (Fig. 1K). The studies suggest that IL2RG is positively associated with the development of PCOS, and it is involved in the inflammatory response of PCOS.

**Table 1:** Characteristics of participants in granulosa cells of patients with PCOS versus controls

	Control	PCOS	*P*-value
**n**	22	18	
**Age (years)**	32.04 ± 0.71	31.95 ± 0.83	0.9058
**BMI (kg/m** ^**2**^ **)**	22.41 ± 0.63	25.79 ± 0.65	0.0006***
**bLH (IU/L)**	4.52 ± 0.34	7.30 ± 0.96	0.0108*
**bFSH (IU/L)**	6.52 ± 0.39	5.25 ± 0.27	0.0053**
**LH/FSH**	0.72 ± 0.06	1.42 ± 0.19	0.0014**
**E2 (pg/ml)**	44.05 ± 3.85	32.55 ± 2.39	0.0206*
**AMH (ng/ml)**	2.62 ± 0.24	7.113 ± 1.18	< 0.0001***
**T (nmol/L)**	0.44 ± 0.05	0.67 ± 0.08	0.045*
**Infertility duration (y)**	2.695 ± 0.43	4.60 ± 0.59	0.0037**
**Number of oocytes**	14.31 ± 1.05	16.67 ± 1.82	0.3904

Data are mean ± SEM. The Mann-Whitney U-test was carried out. bFSH, base of follicle-stimulating hormone; bLH, base of luteinizing hormone; T, testosterone; E2, estradiol; AMH: anti-Müllerian hormone; PCOS, polycystic ovary syndrome. Data are presented as the mean ± SEM, median (interquartile range).

### DHEA-Induced dysfunctions in endocrine and ovarian function in IL2RG KO rats

**Fig. 2:**
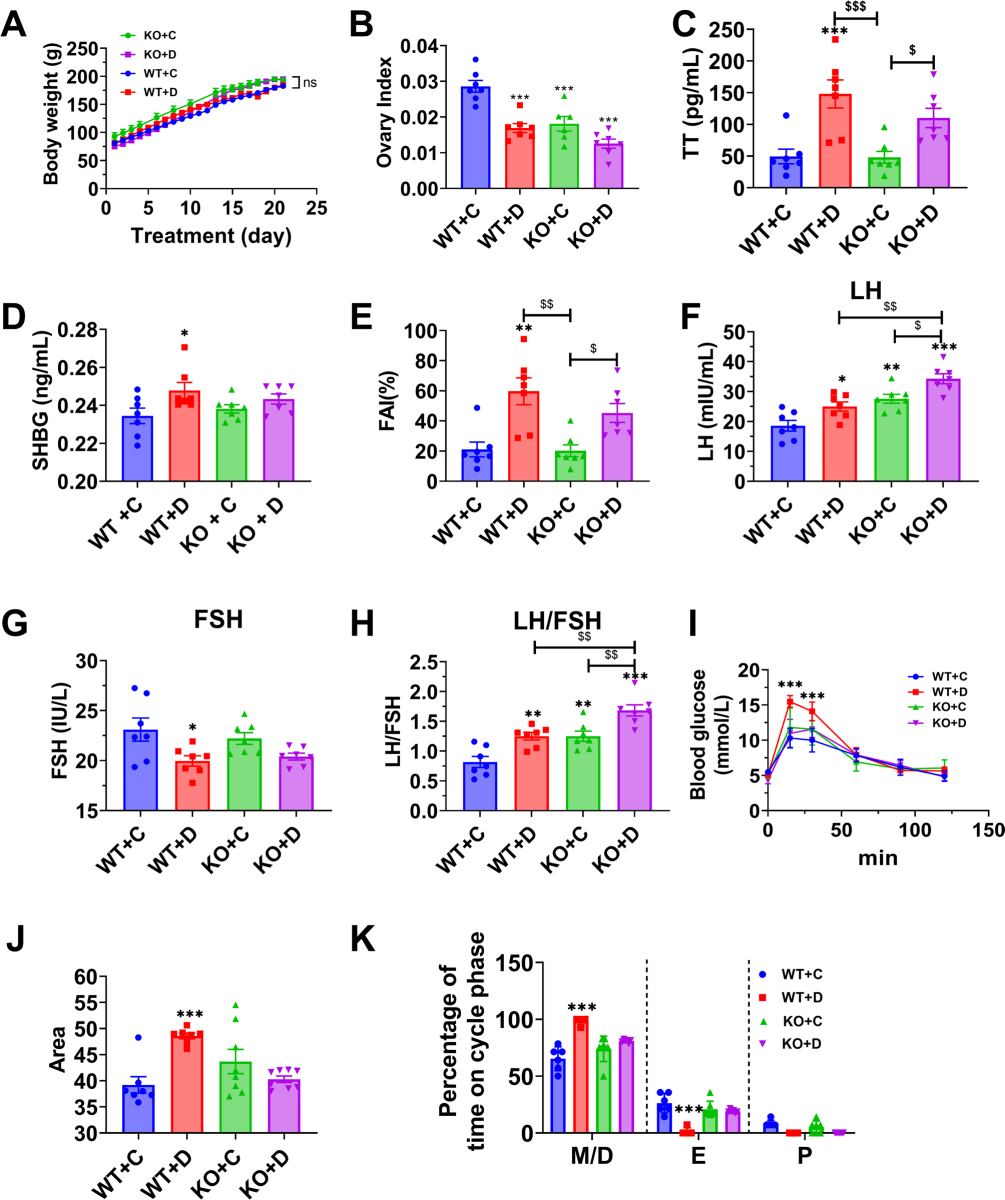
DHEA-induced metabolism and reproductive hormone level disorders in rats. (**A**) Daily bodyweight measurements. (**B**) Ovary index (ovary weight/body weight). (**C**) Total Testosterone (TT) levels. (**D**) Sex Hormone Binding Globulin (SHBG) levels. (**E**) Free androgen index (FAI = T/SHBG*100). (**F**) Serum LH levels. (**G**) Serum FSH levels (**H**) LH/FSH ratio. (**I**) Glucose tolerance test. (**J**) Area under the curve (AUC) of GTT. (**K**) Estrous cycle examination. Indicated stages are proestrus (P), estrus (E), metestrus (M), and diestrus (D). Data are given as mean ± SEM. *n* = 7–8 rats per group. *P* values were determined by one-way ANOVA with Tukey’s multiple comparison post-hoc test. ^$^*P* < 0.05; ^$$^*P* < 0.01. *P < 0.05, **P < 0.01 versus WT + C

To induce PCOS-like model, KO rats were subcutaneously injected with DHEA (6 mg/100 g bodyweight) daily for 21 days in the DHEA group as previously described in the literature, while control rats were injected with corn oil [18, 19]. This model is characterized by elevated androgen levels, increased LH/FSH ratio, cystic follicle development, and disrupted estrous cycles, mimicking key features of human PCOS [20]. Throughout the treatment period, the weight of the DHEA-treated rats remained similar to that of the corn oil-treated rats (Fig. 2A). However, the ovary index (ovary weight/body weight) of DHEA-treated rats was lower than that of the control group (Fig. 2B). The levels of TT, SHBG, free androgen index (FAI), LH, and LH/FSH ratio were elevated in the DHEA-treated rats, except for FSH level (Fig. 2C–H). Figure 2G shows that LH was not increased in the DHEA group. This could be due to the specific model used or the timing of measurement. The increase in LH in the KO group could be attributed to the disruption of normal feedback mechanisms. The KO model might exacerbate this imbalance by altering the hypothalamic-pituitary-gonadal axis [25]. Additionally, compared with corn oil-treated rats, the DHEA-treated rats exhibited abnormal glucose intolerance (Fig. 2I and J) and a reduced proportion of estrus stage (Fig. 2K and sFig. 1 A). However, no significant differences were observed in KO rats. These findings confirm the successful creation of a rat model resembling PCOS, exhibiting abnormalities in metabolism, ovarian structure, and hormonal activity. Furthermore, IL2RG KO demonstrates advantageous effects.

### GSDME is reduced in the ovary of DHEA-Induced KO rats

**Fig. 3:**
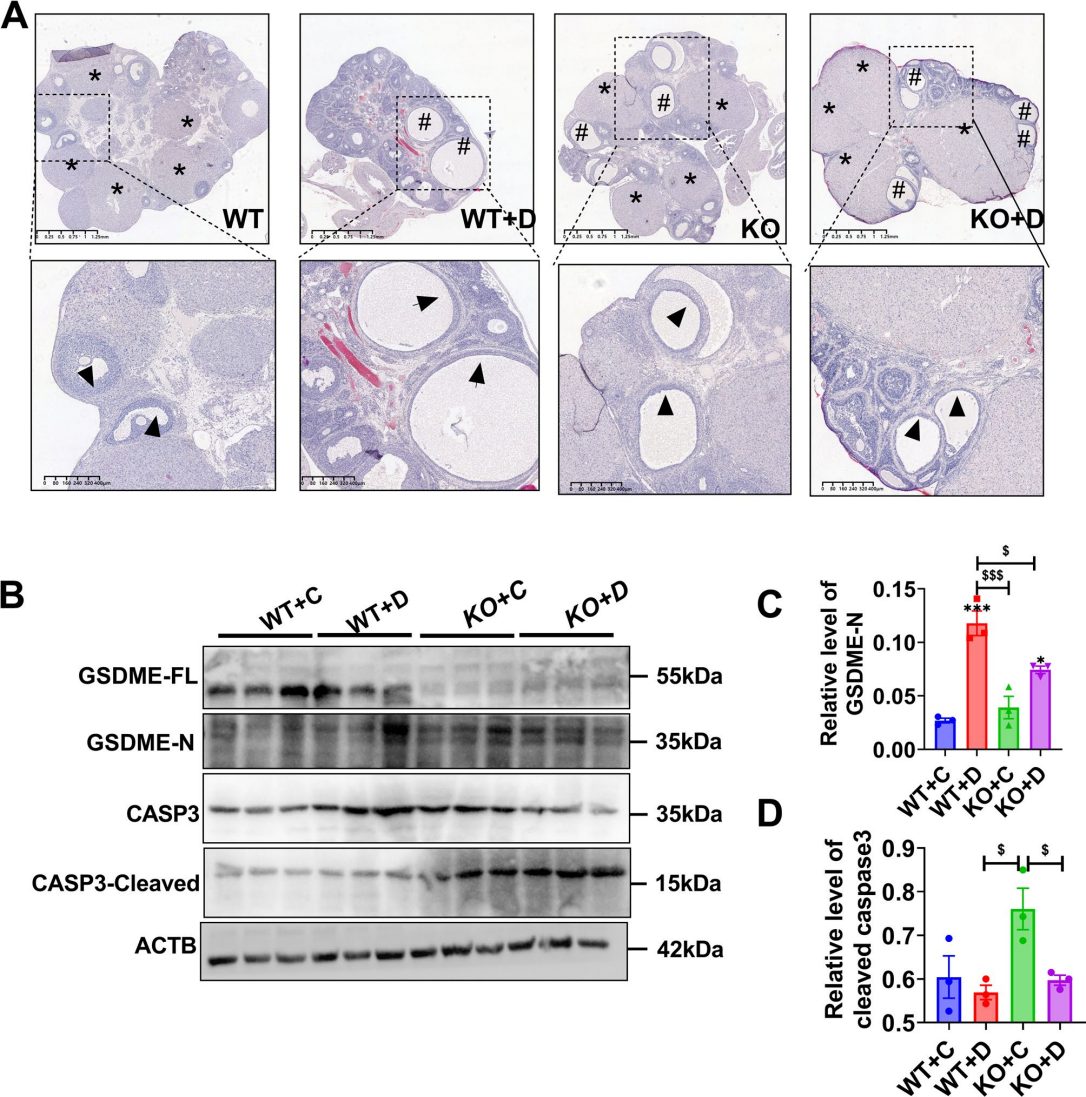
Ovarian morphology and pyroptosis levels in the ovaries of DHEA-induced wild type (WT) and IL2RG KO (KO) rats. (**A**) Hematoxylin and eosin staining of representative ovaries. Scale bar = 1250 μm (up) and 400 μm (down). *: corpus luteum, #: cyst-like follicles; Black arrow: normal granulosa cell layers; Black arrowhead: normal granulosa cell layers. (**B-D**) The levels of pyroptosis-related proteins (GSDME-N/GSDME-FL, Cleaved-CASP3/CASP3,). *P* values were determined by one-way ANOVA with Tukey’s multiple comparison post-hoc test and data are presented as means ± SEM. ^$^*P* < 0.05; ^$$^*P* < 0.01, *P < 0.05, **P < 0.01 versus WT + C

The ovaries of the WT group exhibited follicles at various stages of development, along with several corpora lutea (CL). In contrast, the DHEA group ovaries displayed more cyst-like follicles with collapsed walls and a reduced number of CL (Fig. 3A), indicating a resemblance to a rat model resembling PCOS. The expression of GSDME-N protein was significantly increased in DHEA-treated rat, but decreased in DHEA-treated KO rats (Fig. 3C). Additionally, the levels of cleaved-CASP3 were significantly increased (Fig. 3D). Hyperandrogenic stimulation elicits GSDME-dependent pyroptosis in the ovary. Conversely, upon the knockdown of IL2RG, the extent of pyroptosis was decreased, while the expression level of the apoptotic cleavage product, CASP3, was increased. These findings indicate that upon the knockdown of IL2RG, a fraction of the pyroptotic process is converted into the apoptotic pathway. Ovarian follicles in KO rats treated with DHEA showed a pronounced tendency to undergo apoptosis compared to those in KO rats. This shift may have significant implications for the overall health and function of ovarian follicles affected by PCOS.

### **CASP3 Cleaves GSDME and Inhibits T-Induced Pyroptosis in KGN Cells In Vitro**.

**Fig. 4:**
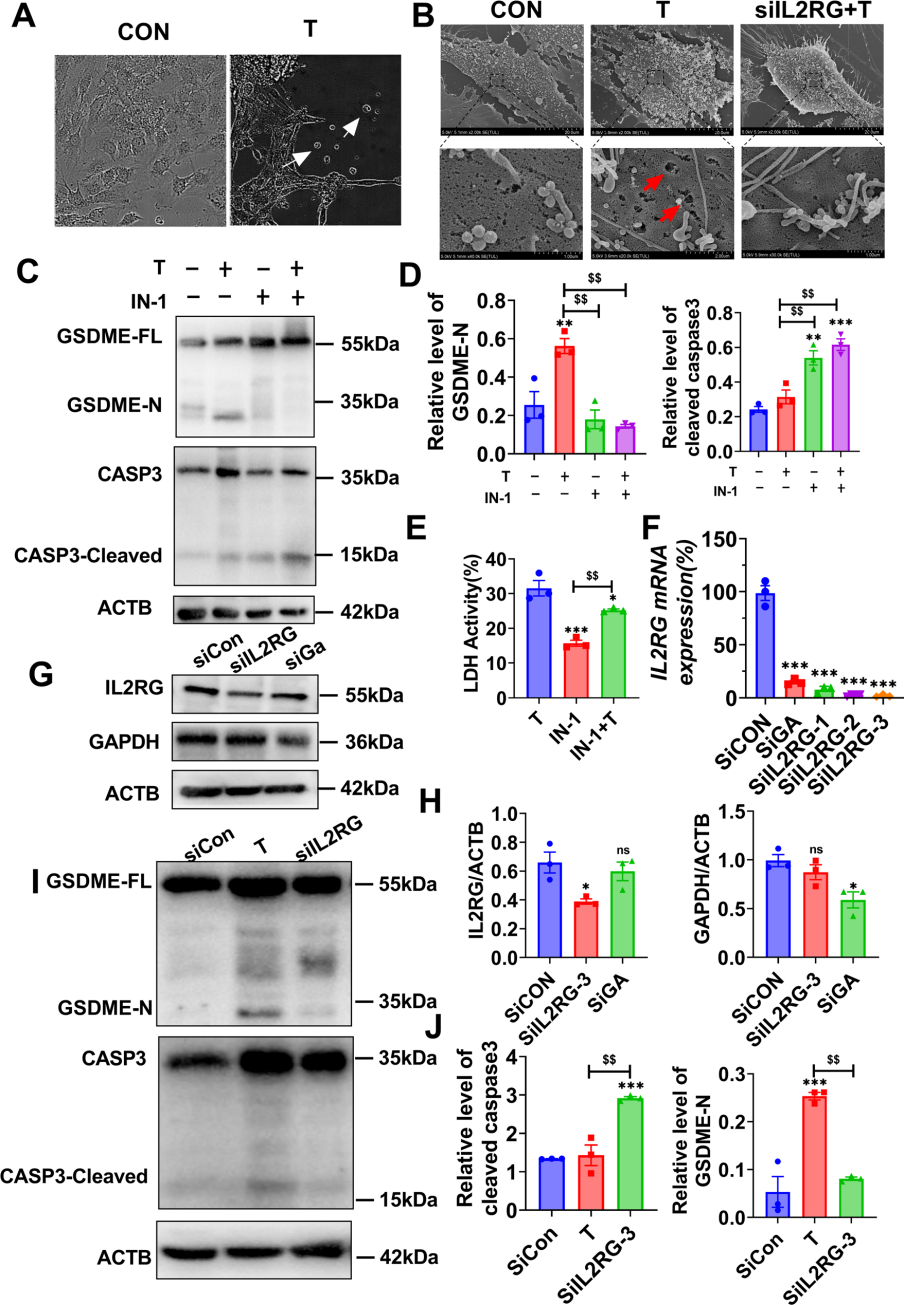
IL2RG deletion inhibits T-induced pyroptosis in vitro. (**A**) The morphology of granulosa cells was assessed by optical microscopy and the white arrows show the cell swelling and roundness. (**B**) Scanning Electron Microscope (SEM): red arrows show many prominent vesicles on the cell surface that are characteristic of pyroptosis. (**C**) Western blot analysis was performed to assess the cleavage of GSDME by caspase-3 in KGN cells treated with STAT5-IN-1 (IN-1) and testosterone. CASP3: Caspase-3; CASP3-cleaved: Cleaved-Caspase-3; FL: full-length; GSDME-N: the N-terminal cleavage products of GSDME, respectively. (**E**) LDH levels. (**F)** qRT-PCR of *IL2RG* knockdown efficiency by siRNA. (**G** and **H**) WB assays showed the knockdown efficiency of IL2RG in KGN cells. (**I** and **J**) WB of siIL2RG and T-induced KGN cells GSDME cleavage by CASP3 and GSDME-N respectively. *P* values were determined by one-way ANOVA with Tukey’s multiple comparison post-hoc test and data are presented as means ± SEM. For F and H, *P* values were determined by ANOVA followed by Dunn’s post-hoc test. **P < 0.01 versus Column A. ^$^*P* < 0.05; ^$$^*P* < 0.01

To investigate the relationship between low-grade inflammation and pyroptosis following T induction in vitro, we cultured KGN cells with T for up to 24 h and observed the presence of “puffed” cells under microscopy (Fig. 4A), as well as increased efflux of cellular contents and prominent vesicles in the T-induced group through SEM (Fig. 4B). Hyperandrogenism stimulates chronic low-grade inflammation in the ovary and induces pyroptosis through GSDMD cleavage [14, 26]. Studies have shown that IL2RG couples to JAK tyrosine kinases and activates STAT5 transcription factors [27]. Given that STAT5 can be part of the IL2RG receptor complex and is crucial for the signaling of many cytokines relevant to PCOS, its inhibition by IN-1 could reduce IL2RG signaling [28, 29]. To further investigate the function of IL2RG, we first added STAT5-IN-1 (IN-1), a STAT5 inhibitor, to inhibit IL2RG for 3 h and then added T for 24 h. As a result, the level of GSDME decreased, while the cleaved-CASP3 increased (Fig. 4C and D). Furthermore, the release of LDH in IN-1-induced cells was significantly reduced, indicating that T-induced cell death involves at least two distinct modes, one of which is suppressed upon knockdown of IL2RG expression (Fig. 4E). Although IL2RG activation leads to STAT5 phosphorylation, data of IN-1 (STAT5 inhibitor) is not sufficient to indicate the role of IL2RG [30]. Subsequently, recombinant small interfering RNAs (siRNAs) targeting *IL2RG* were administered, with siGAPDH acting as a positive control. More than 50% of *IL2RG* was knocked down in KGN cells (Fig. 4F and H), and the knockdown cells displayed lower levels of GSDME-N after T treatments. Moreover, the CASP3 cleavage was not decreased and even increased compared to the control (Fig. 4I and J). These findings demonstrate the coexistence of apoptosis and pyroptosis in T-induced KGN cells, with a notable inhibition of pyroptosis upon knockdown of *IL2RG*.

### Disruption of IL2RG skews KGN cell death from pyroptosis to apoptosis

**Fig. 5:**
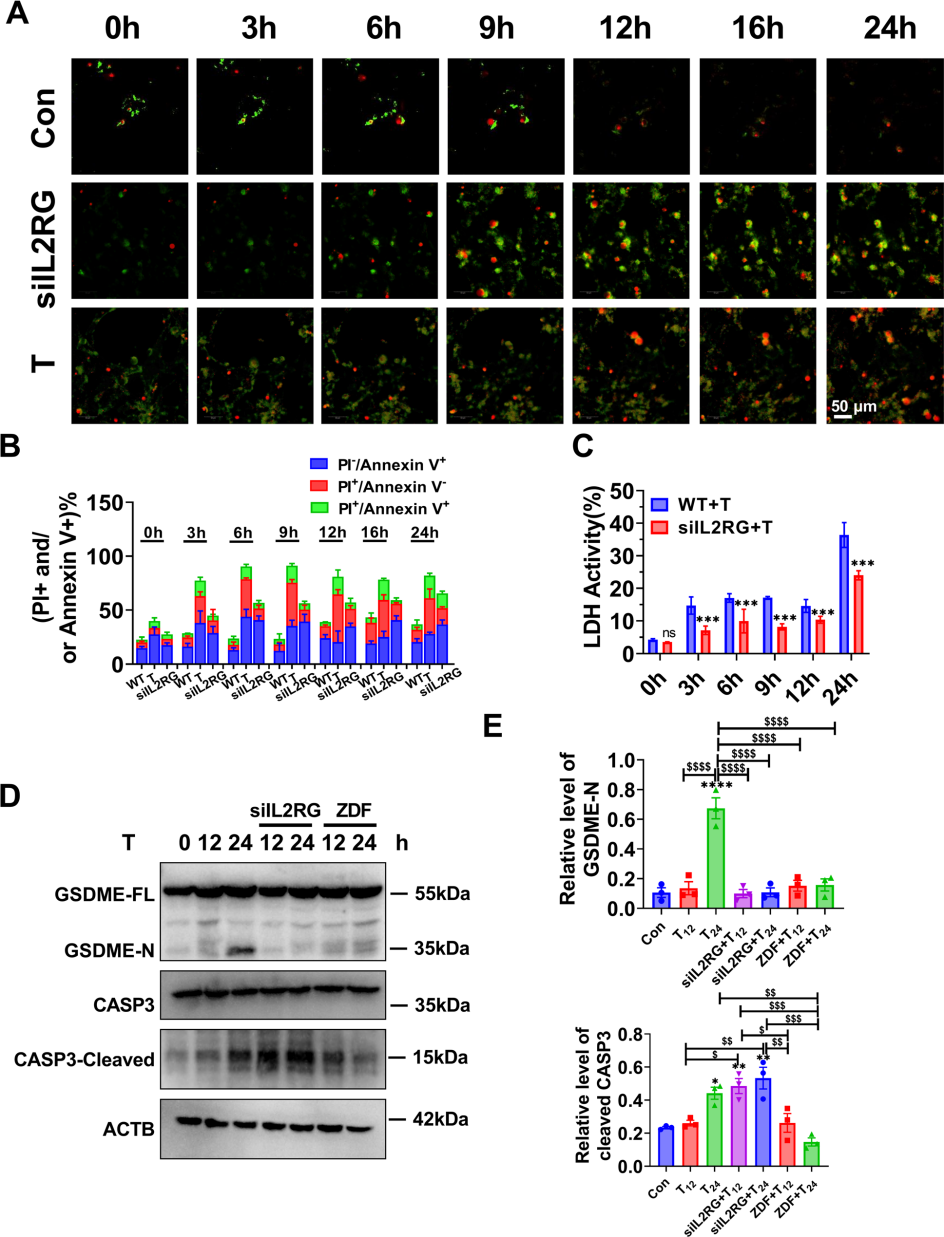
IL2RG disruption skews GSDME-mediated pyroptosis to apoptosis. (**A**) KGN cells were incubated with annexin V-FITC and PI and the cells were imaged by fluorescence microscopy (scale bar = 50 μm). (**B**) The percentage of AV^−^PI^−^, AV^+^PI^−^, and AV^+^PI^+^ cells at the indicated time points. (**C**) LDH levels. (**D-E**) The levels of pyroptosis-related proteins (cleaved-CASP3, GSDME-N/GSDME). T_12_: KGN treated with T for 12 h; KO_12_: IL2RG KO group treated with T for 12 h; ZDF12: ZDF pre-treated for 3 h and then treated with T for 12 h. *n* = 3 independent repeats. *P* values were determined by one-way ANOVA with Tukey’s multiple comparison post-hoc test and data are presented as means ± SEM. **P < 0.01 versus Column A. ^$^*P* < 0.05; ^$$^*P* < 0.01

**Fig. 6:**
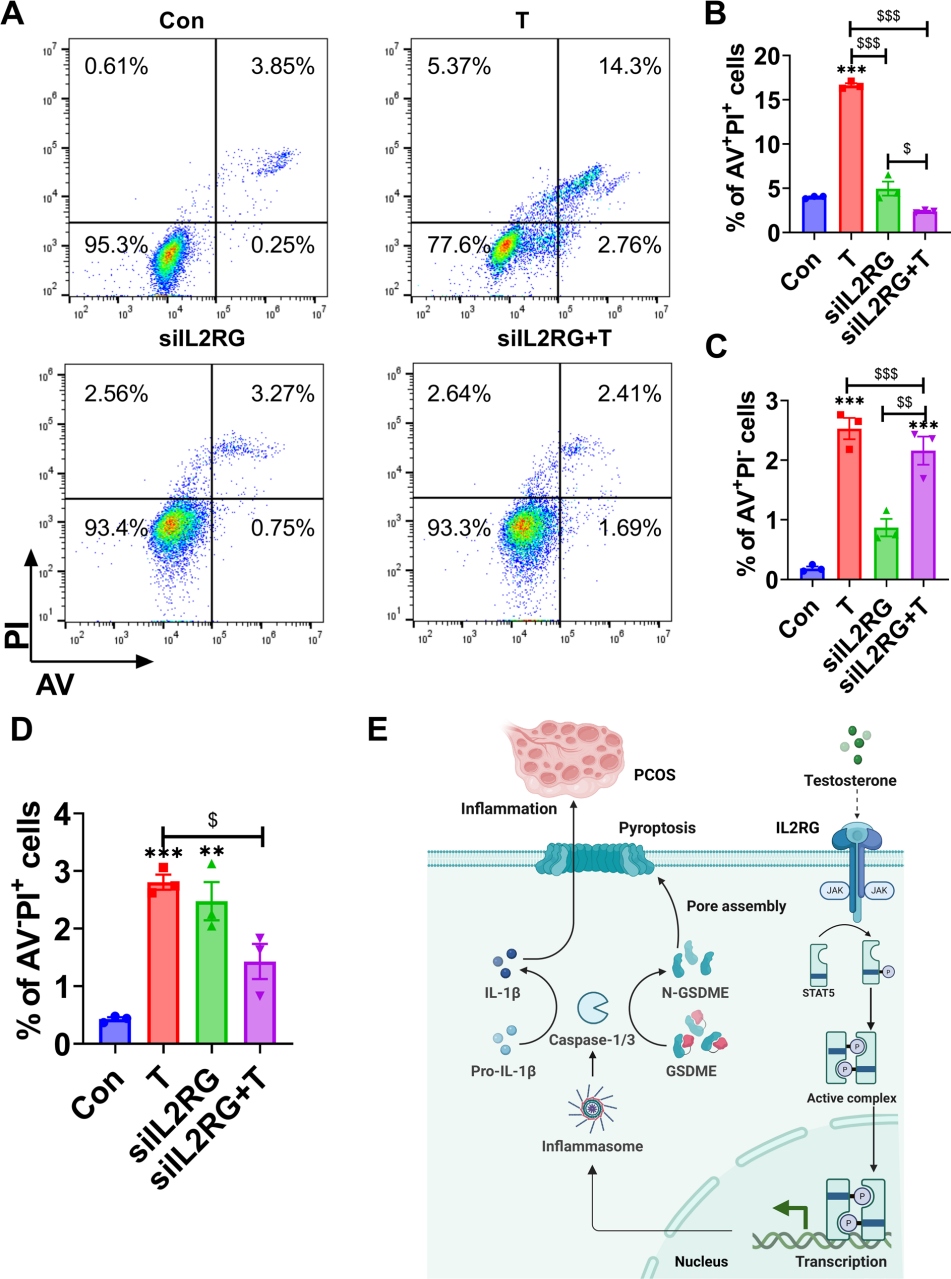
Representative flow cytometry plots of KGN cells undergoing pyroptosis. (A-D) The percentage of AV^+^PI^+^, AV^+^PI^−^ and AV^−^PI^+^ cells at the indicated time points. (E) Graphic abstract illustrating the role of IL2RG in PCOS. Created in https://BioRender.com. *n* = 3 independent repeats. P values were determined by one-way ANOVA with Tukey’s multiple comparison post-hoc test and data are presented as means ± SEM. **P < 0.01 versus Column A. ^$^*P* < 0.05; ^$$^*P* < 0.01

To further investigate the impact of CASP3 on T-induced cell death in KGN cells, a time-course study was conducted to examine the effects of T treatment (Fig. 5A). The Annexin V/PI double staining assay was employed. Notably, both the apoptotic and pyroptotic cell populations contained PI^+^ cells. In terms of morphology, the sizes of all PI^+^ cells were found to be reduced, and the fluorescence intensity was increased in the *IL2RG*-knockdown cells compared to PI^+^ T-induced cells. Given that one of the major differences between pyroptosis and apoptosis is the integrity of the plasma membrane, the PI^+^ cells at an early time point might represent pyroptosis better than apoptosis. The proportions of PI^+^ cells were significantly earlier and higher in the T-induced group compared to the control and *IL2RG*-knockdown groups (Fig. 5B). Notably, the percentage of PI^+^ cells decreased in the *IL2RG*-knockdown group compared with the T group. The earlier time point of PI^+^ cells and the lower proportion of PI^+^ cells indicate a transition from T-induced pyroptosis to apoptosis. Additionally, T-induced *IL2RG*-knockdown cells exhibited significantly reduced LDH release, suggesting that LDH release is downstream of GSDME cleavage (Fig. 5C). To further confirm the switch from apoptosis to pyroptosis, T-induced control and *IL2RG*-knockdown KGN cells were treated with the specific CASP3 inhibitor ZDF. ZDF significantly suppressed GSDME cleavage, suggesting that CASP3 cleaved GSDME. The level of cleaved-CASP3 in the *IL2RG*-knockdown group was higher than in the T-induced group, further indicating that knockdown of *IL2RG* induced a shift from pyroptosis to apoptosis (Fig. 5D-E). Furthermore, the flow cytometry analysis of cell death exhibited a high degree of concordance with the observations made through microscopy. The number of AV^+^PI^+^ cells was lower in the *IL2RG*-knockdown group than in the control group (Fig. 6A-D). These findings are consistent with the idea that GSDME specifically requires CASP3 to switch T-induced apoptosis to pyroptosis.

Potential interactions between IL2RG and GSDME predicted by the STRING website in this study revealed that IL2RG and GSDME were linked through IL-1β (sFig. 2B). The expression of IL-1β was found to be lower in the KO group and higher in the WT + D group (sFig. 2 C). To further explore the connection between IL2RG and GSDME, we used TIMER2 database to perform a Spearman correlation analysis and observed a positive correlation between GSDME and IL2RG (*r* = 0.88) in various cancers (sFig. 2D). This suggests a potential indirect mechanism whereby IL2RG activation leads to increased GSDME (DFAN5), subsequently enhancing GSDME-mediated pyroptosis. Additionally, GTEx (in GEPIA database) analysis results revealed a positive Pearson correlation between IL2RG and GSDME (*R* = 0.22) in ovary (sFig. 2E).

## Discussion

In this study, IL2RG KO exerts an inhibitory effect on CASP3-mediated GSDME cleavage and redirects pyroptosis towards apoptosis. The knockdown of *IL2RG* can convert T-induced apoptosis to pyroptosis in KGN cells. These results suggest a model shown in Fig. 6B.

Mutation of the *IL2RG* gene has been demonstrated to cause X-linked severe combined immune deficiency (XSCID) in humans [31, 32]. Studies had reported endometriosis was induced in BALB/c-Rag2(-/-) IL2RG (-/-) mice by surgical implantation of human endometrial fragments [33]. IL2RG knockout mice showed less regularity in estrous cycle and one of the IL2RG-dependent cytokines, IL-15, was implicated in the pathogenesis of PCOS [7, 34]. The above results indicate that the ovarian function of IL2RG knockout (KO) mice is disrupted, and it can induce estrogen-related diseases. However, to date, no prior investigations had investigated the reproductive function of IL2RG in PCOS. Consequently, we enrolled PCOS patients who exhibited typical PCOS-associated characteristics, including elevated BMI, an increased LH to FSH ratio, higher testosterone levels, elevated AMH levels, and a prolonged duration of infertility (Table 1). It has been reported that infertility in PCOS patients is frequently linked to a higher BMI and a long infertility duration ^[1]^. Thus, the patients in this study conformed to the diagnostic criteria for PCOS. Although polycystic ovarian morphology is a hallmark of PCOS, it does not invariably imply that the number of retrieved oocytes in PCOS patients will be greater than that in control subjects. Our results showed the evaluated expression level of IL2RG in human GCs and IL2RG-dependent cytokines (IL-2, IL-4, IL-15) in human follicular fluid. Although reporters reported one of the IL2RG-dependent cytokines, IL-2, levels decreased in the endometrial fluid in women with PCOS [35], it is important to note that our current study focused on the follicular fluid, which reflects the local cytokine environment within the ovary. Given the distinct physiological functions and immune environments of these two compartments, it is plausible that IL-2 levels may be differentially regulated in the ovaries and uterus of PCOS patients [36].

The IL-2, 4, 7, 9, 15, and 21 systems may affect the regulation of estrous cycles at the hypothalamic-pituitary level [37, 38]. PCOS patients were characterized by excessive androgen secretion and aberrant follicular development [39]. This disruption may affect the secretion of gonadotropin-releasing hormone, LH, and FSH. The increase in LH in the KO group could be attributed to the disruption of normal feedback mechanisms. The KO model might exacerbate this imbalance by altering the hypothalamic-pituitary-gonadal axis. The observed changes in LH and FSH levels may reflect alterations in follicular development and steroid hormone production in the ovaries of KO and DHEA-treated rats. These hormones are related to metabolic disorders, such as diabetes and insulin resistance, overweight and obesity, infertility, and disturbed menstrual cycle in PCOS patients [40]. Furthermore, our immunohistochemical findings in Fig. 1K confirm that IL2RG knockout primarily affects ovarian somatic cell function rather than immune cell populations, establishing IL2RG as a novel ovarian-specific target in PCOS and ruling out global immune dysregulation as the primary pathogenic mechanism. Although Fig. 2F shows that there is no statistical difference in luteinizing hormone (LH) in DHEA-treated group, there is a tendency for LH to increase. Moreover, the ratio of luteinizing hormone (LH) to follicle-stimulating hormone (FSH) has a statistically significant increase (Fig. 2H).

Hyperandrogenism and chronic inflammation are considered the main causes of PCOS, but the molecular mechanisms have not been fully clarified [41]. A study demonstrates that PCOS mice exhibit enhanced macrophage accumulation and up-regulation of genes associated with inflammatory response, potentially impacting cardiovascular health in women [3]. Pyroptosis is widely acknowledged as a type of programmed cell death mediated by gasdermin, characterized by necrosis and inflammation. In PCOS females and animal models, there is an observed correlation between chronic inflammatory processes and increased levels of various proinflammatory cytokines and chemokines. Pyroptosis with GSDME oligomerization and complete liberation of IL-1β is similar to GSDMD pyroptosis [42]. This inflammatory dysfunction may affect signaling pathways within cells and indirectly regulate the expression or activity of GSDME. These results indicate that the presence of proinflammatory factors in PCOS mice contributes to the induction of pyroptotic cell death in ovarian cells, thereby disrupting follicular development.

In this study, GSDME is cleaved and activated specifically by CASP3, and this cleavage causes pyroptosis. While we recognize the importance of caspase-1 in canonical pyroptosis involving GSDMD [43], we detected the expression level of pyroptosis in the following groups: Con, T, ZDF (Z-DEVD-FMK, caspase3 inhibitor) and VX-657 (Belnacasan, caspase-1 inhibitor). The results showed that the VX-765 was less effective in inhibiting GSDME-mediated pyroptosis than the ZDF (sFig. 1B-C).

Our research employed a caspase-3 inhibitor (ZDF) in cellular assays, along with a caspase-1 inhibitor VX-657 in KGN cells; the data from cellular and in vivo DHEA-induced PCOS mice showed that IL2RG regulates pyroptosis via dual caspase pathways and is crucial in linking metabolic stress to PCOS pathogenesis.

In a prior study, elevated levels of IL-1β were observed in hyperandrogenism-induced PCOS-like mice [26]. Analysis using the STRING database suggests potential interactions between IL2RG and GSDME, mediated through IL-1β, indicating a linkage between these two molecules. Furthermore, the relationship between GSDME and IL2RG across various cancers was corroborated by the TIMER2 database, hinting at a potential indirect mechanism in which IL2RG activation may enhance GSDME-mediated pyroptosis. Additionally, GTEx data (accessed via the GEPIA database) revealed a connection between IL2RG and GSDME in ovarian tissue, although this correlation was weaker compared to the findings in TIMER2.

While pyroptosis was suggested to be involved in the hyperandrogen-induced reverse effects (disruption of the endocrine system and inflammatory response in animal studies), our findings offer new targets to achieve the transformation between hyperandrogen-induced pyroptosis and apoptosis. We hypothesized that blocking STAT5 phosphorylation could offer initial insights into the potential role of IL2RG. To overcome this limitation and establish more direct evidence of the specific involvement of IL2RG, we conducted IL2RG knockdown experiments. These knockdown experiments revealed that IL2RG knockdown directly influenced GSDME cleavage and enhanced apoptosis in cells, offering robust and specific evidence supporting the contribution of IL2RG to the observed phenotype. Additional control groups (T - treated and siIL2RG) were incorporated to clearly determine whether the observed suppression of T-induced pyroptosis in KGN cells is due to IL2RG knockdown or T absence, and our data clarify the role of IL2RG in relation to T-mediated effects. Moreover, IL2RG knockdown inhibited T-induced pyroptosis, resulting in the transition from pyroptosis to apoptosis via inhibition of CASP3 activation. The transition from pyroptosis to apoptosis was validated by assessing GSDME-N and cleaved CASP3 levels at 12 h and 24 h time points in our in vitro experiments. Notably, the 24 h time point exhibited a more pronounced effect, highlighting a stronger shift in the trajectory of this cellular process.

This shift from pyroptosis to apoptosis upon IL2RG knockdown suggests a potential therapeutic strategy for PCOS. Pyroptosis is a pro-inflammatory form of cell death, releasing DAMPs (Damage-associated molecular patterns) that exacerbate the inflammatory environment in PCOS ovaries. By shifting the cell death pathway towards apoptosis, which is less inflammatory, we may be able to reduce the local inflammation and improve ovarian function [44]. For instance, a combined therapy targeting IL2RG with an anti-inflammatory agent may synergistically reduce PCOS symptoms. Further research exploring how IL2RG influences cellular pathways related to inflammation and cell death could potentially lead to novel treatment strategies aimed at restoring normal ovarian function in individuals with PCOS.

Our findings support the growing recognition of chronic low-grade inflammation as a key driver of PCOS pathogenesis. IL2RG-dependent cytokines likely contribute to this inflammation, and our data suggests that this inflammation promotes pyroptosis in granulosa cells, further exacerbating the condition. The IL2RG-GSDME axis may represent a novel inflammatory pathway in PCOS, distinct from or acting in concert with previously identified pathways [11, 42, 45]. In PCOS, the IL2RG/GSDME axis interacts with key pathways: hyperandrogenism signals through IL-2R, activating CASP3 which cleaves GSDME. The N-terminal GSDME fragment triggers pyroptosis, releasing IL-1β, thus amplifying inflammation. CASP3 also independently initiates apoptosis. Therefore, this axis connects hyperandrogenism, inflammation via IL-1β, and cell death, contributing to PCOS pathology. Targeting IL2RG could offer a new therapeutic avenue by modulating both cytokine signaling and cell death pathways.

Our research employed DHEA-induced PCOS mice in in vivo experiments and a caspase-3 inhibitor (Z-DEVD-FMK, ZDF) in cellular assays, along with a caspase-1 activity assay in KGN cells; cellular and in vivo data showed that IL2RG regulates pyroptosis via dual caspase pathways and is crucial in linking metabolic stress to PCOS pathogenesis.

In conclusion, KO female rats exhibit attenuation of DHEA-induced PCOS pathogenesis by shifting cell death from pyroptosis to apoptosis through the GSDME pathway and offer potential for the detection and treatment of PCOS. Understanding the molecular mechanisms underlying the involvement of IL2RG in PCOS can provide valuable insights into potential therapeutic targets for this complex disorder.

## Conclusion

In PCOS, IL2RG is expressed at higher levels in both GCs and theca cells. The elevated expression of IL2RG in PCOS has significant implications within ovarian cells. One notable effect is its inhibitory action on CASP3-mediated GSDME cleavage upon knockdown. By inhibiting CASP3-mediated GSDME cleavage, IL2RG redirects the fate of pyroptotic cell death towards apoptosis. In conclusion, KO female rats exhibit attenuation of DHEA-induced PCOS pathogenesis by shifting cell death from pyroptosis to apoptosis through the GSDME pathway and offer potential for the detection and treatment of PCOS. Understanding the molecular mechanisms underlying the involvement of IL2RG in PCOS can provide valuable insights into potential therapeutic targets for this complex disorder.

## Supplementary Information

Below is the link to the electronic supplementary material.

Supplementary Material 1

## Data Availability

No datasets were generated or analysed during the current study.
