# Supplementary material for: IL2RG knockout mitigates polycystic ovary syndrome pathogenesis by transitioning pyroptosis to apoptosis through the GSDME pathway
Source: J Ovarian Res. 2025 Aug 19;18:189. doi: 10.1186/s13048-025-01774-4 (PMC12362916; doi:10.1186/s13048-025-01774-4)
Supplement: Supplementary file 1 — Supplementary Material 1 [file 13048_2025_1774_MOESM1_ESM.docx]

**
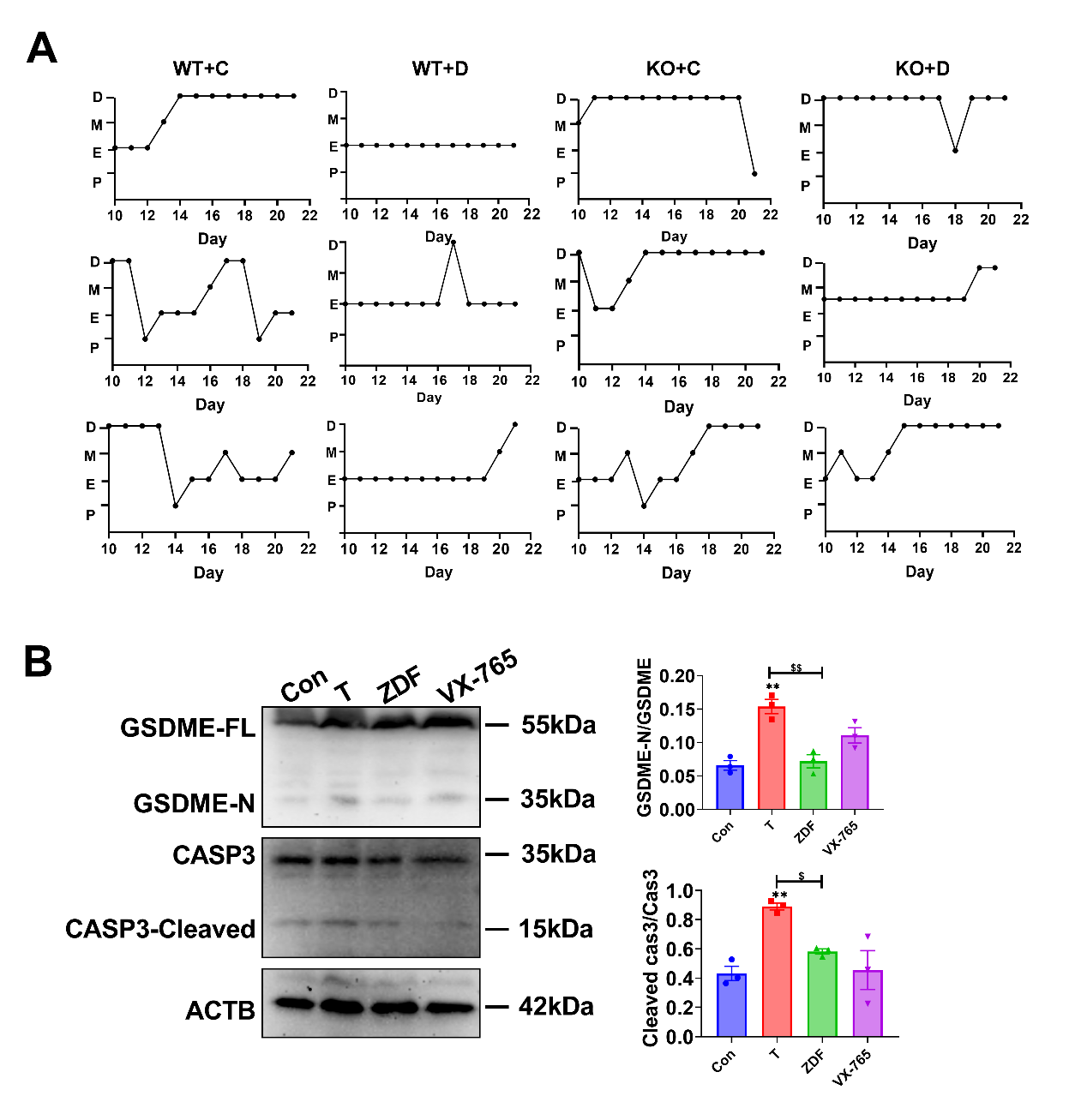
**

**sFigure 1 (A)** Representative estrous cycles. n=3 **(B)** Western blot analysis was performed to assess the IL-1β expression level in rats.


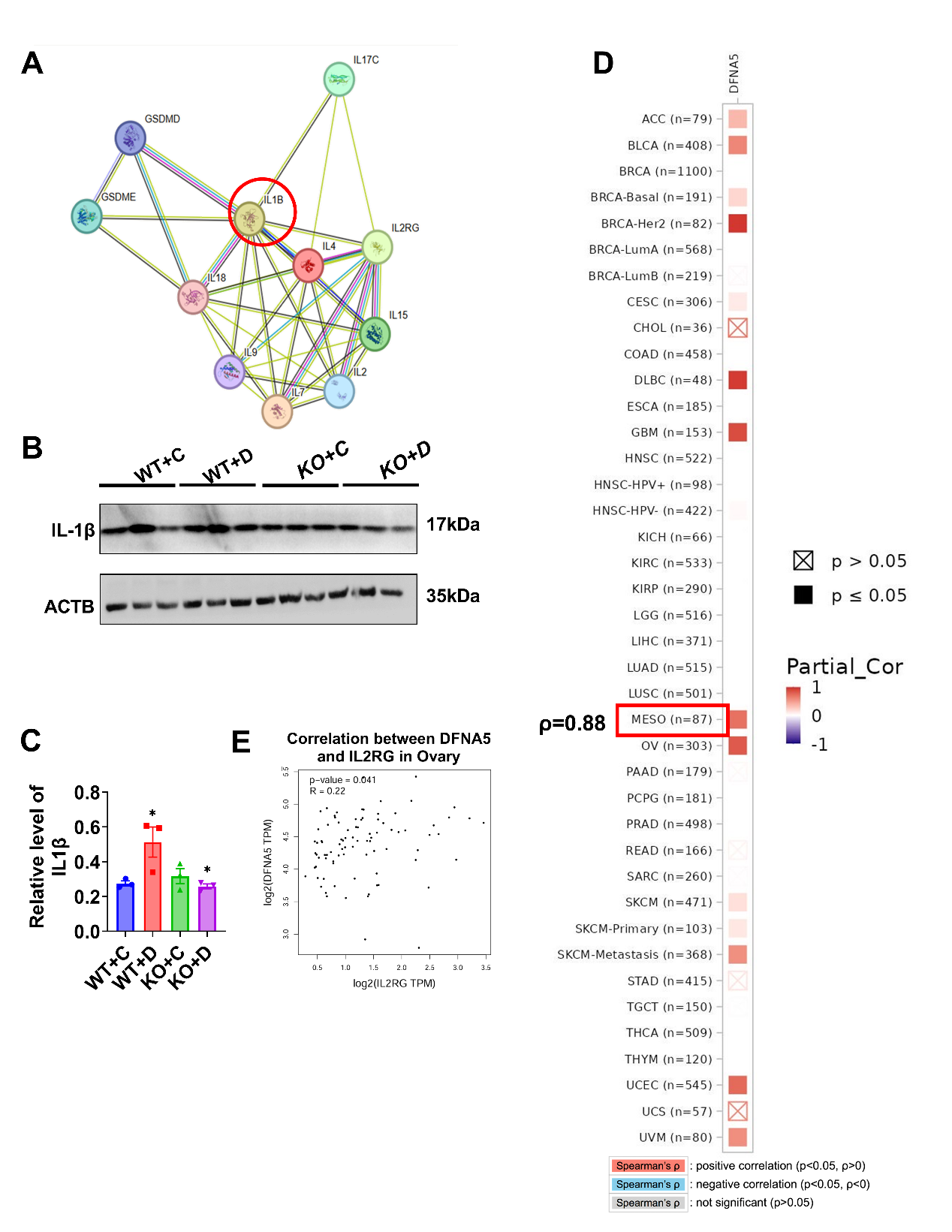


**sFigure 2** The connection between IL2RG and GSDME (DFNA5). (A) Prediction of endogenous interaction between IL2RG and other proteins using the STRING website (https://string-db.org). (B-C) Western blot analysis was performed to assess the IL-1β expression level in rats and WB quantification. (D) Correlations between the DFNA5 gene and IL2RG in various cancer types using https://compbio.cn/timer2. (E) Partial correlations between the DFNA5(GSDME) gene and IL2RG from the GEPIA database (http://gepia.cancer - pku.cn/detail.php?clicktag = correlation)
